# EphrinB1: novel microtubule associated protein whose expression affects taxane sensitivity

**DOI:** 10.18632/oncotarget.2823

**Published:** 2014-11-26

**Authors:** Paul L. Colbert, Daniel W. Vermeer, Bryant G. Wieking, John H. Lee, Paola D. Vermeer

**Affiliations:** ^1^ Cancer Biology Research Center, Sanford Research, Sioux Falls, South Dakota, USA

**Keywords:** EphrinB1, taxane, mitosis, microtubule, PTPN13

## Abstract

Microtubules (MTs) are components of the cytoskeleton made up of polymerized alpha and beta tubulin dimers. MT structure and function must be maintained throughout the cell cycle to ensure proper execution of mitosis and cellular homeostasis. The protein tyrosine phosphatase, PTPN13, localizes to distinct compartments during mitosis and cytokinesis. We have previously demonstrated that the HPV16 E6 oncoprotein binds PTPN13 and leads to its degradation. Thus, we speculated that HPV infection may affect cellular proliferation by altering the localization of a PTPN13 phosphatase substrate, EphrinB1, during mitosis. Here we report that EphrinB1 co-localizes with MTs during all phases of the cell cycle. Specifically, a cleaved, *un*phosphorylated EphrinB1 fragment directly binds tubulin, while its phosphorylated form lacks MT binding capacity. These findings suggest that EphrinB1 is a novel microtubule associated protein (MAP). Importantly, we show that in the context of HPV16 E6 expression, EphrinB1 affects taxane response *in vitro*. We speculate that this reflects PTPN13's modulation of EphrinB1 phosphorylation and suggest that EphrinB1 is an important contributor to taxane sensitivity/resistance phenotypes in epithelial cancers. Thus, HPV infection or functional mutations of PTPN13 in non-viral cancers may predict taxane sensitivity.

## INTRODUCTION

Mitosis is a complex sequence of highly regulated events that ensures the proper segregation of sister chromatids into daughter cells. At the heart of the mitotic machinery lie the microtubules (MTs), components of the cytoskeleton made up of polymerized alpha and beta tubulin dimers [1]. MTs critically modulate cell shape, structure and movement during interphase and elaborate the mitotic spindle at mitosis. Thus, MT structure and function must be maintained throughout the cell cycle. Moreover, their contribution to such a diverse array of cellular functions requires strict regulation. As such, alpha and beta tubulin are subjected to a host of post-translational modifications (e.g. tyrosination/detyrosination, glutamylation, glycylation, actetylation/deacetylation, and phosphorylation ([2] [3] [4]) that generate distinct subpopulations of MTs; some MTs uniformly modified throughout, while others display various modifications peppered along their length. Such variability in extent and composition of tubulin modifications translates into a rich complexity that dictates MT function, stability and associations with microtubule associated proteins, or MAPs [2]. MAPs directly bind tubulin and themselves undergo modifications that regulate their functions and associations. For example, phosphorylation of MAPs functions as an on/off switch such that when phosphorylated, MAPs lose their association to MTs [5-7]. In fact, phosphorylation is an important example of how kinases and phosphatases regulate mitosis. However, while much is understood regarding the function of kinases during mitosis, much less is clearly defined regarding the opposing functions of phosphatases [8-15].

The protein tyrosine phosphatase PTPN13 (PTPBL is the murine ortholog) localizes at centrosomes from interphase through metaphase and dramatically shifts to the spindle midzone during anaphase. At telophase, PTPN13 accumulates at the midzone, concentrating at the center of the midbody during cytokinesis. PTPN13's localization to specific sites along the mitotic spindle suggests that it regulates distinct aspects of mitosis and/or cytokinesis [16-18]. Interestingly, decreased PTPN13 expression correlates with changes in cellular proliferation and invasive characteristics in multiple epithelial cells *in vitro* as well as in tumors *in vivo.* Taken together, these data suggest not only that PTPN13 is a tumor suppressor, but also that loss of PTPN13 expression or function affects mitosis [19-27]. Its localization pattern throughout the cell cycle is consistent with this idea and suggests that PTPN13 may directly regulate mitosis.

PTPN13's five PDZ domains mediate associations with a variety of different cellular components [17, 18]. One of these PTPN13 binding partners, EphrinB1, is also a phosphatase substrate. EphrinB1 belongs to a family of ligands which bind and activate Eph receptor tyrosine kinases [28]. Ephrin ligands are unique; following Eph receptor engagement, Ephrins themselves become activated and initiate their own downstream signaling termed “reverse signaling” [29, 30]. Moreover, recent studies suggest that Ephrin ligands play a role in oncogenesis and/or metastasis [28, 31, 32]. Given PTPN13's localization to distinct compartments during mitosis/cytokinesis together with newly appreciated functions of Ephrin ligands in oncogenesis/metastasis, we hypothesized that EphrinB1 contributes to the processes of mitosis and/or cytokinesis where PTPN13 stands poised to regulate its activation. Here, we report that EphrinB1 co-localizes with MTs during all phases of the cell cycle. Specifically, an *un*phosphorylated cleaved fragment of EphrinB1 directly binds tubulin. Interestingly, phosphorylated EphrinB1 appears to be excluded from MT structures. Upon mitotic entry, EphrinB1 localizes to the centrosomes and mitotic spindle from prophase through telophase. At cytokinesis, EphrinB1 localizes to the midbody except at the point immediately at the center, where PTPN13 resides [16]. This novel description of EphrinB1 localization, its tubulin binding capacity and documented roles in carcinogenesis are consistent with functional regulation of mitosis. Importantly, we questioned whether increased expression of EphrinB1 correlates with sensitivity to taxanes while its absence confers resistance. Finally, we examined if EphrinB1 also localized to mitotic figures in human cancer samples. Together, these data will elucidate the role EphrinB1 plays as a novel MAP and its potential implications into responses to anti-microtubule therapy agents like taxanes.

## RESULTS

### EphrinB1 immunolocalizes in a microtubule-like pattern and co-localizes with alpha and beta tubulin

**Figure 1:**
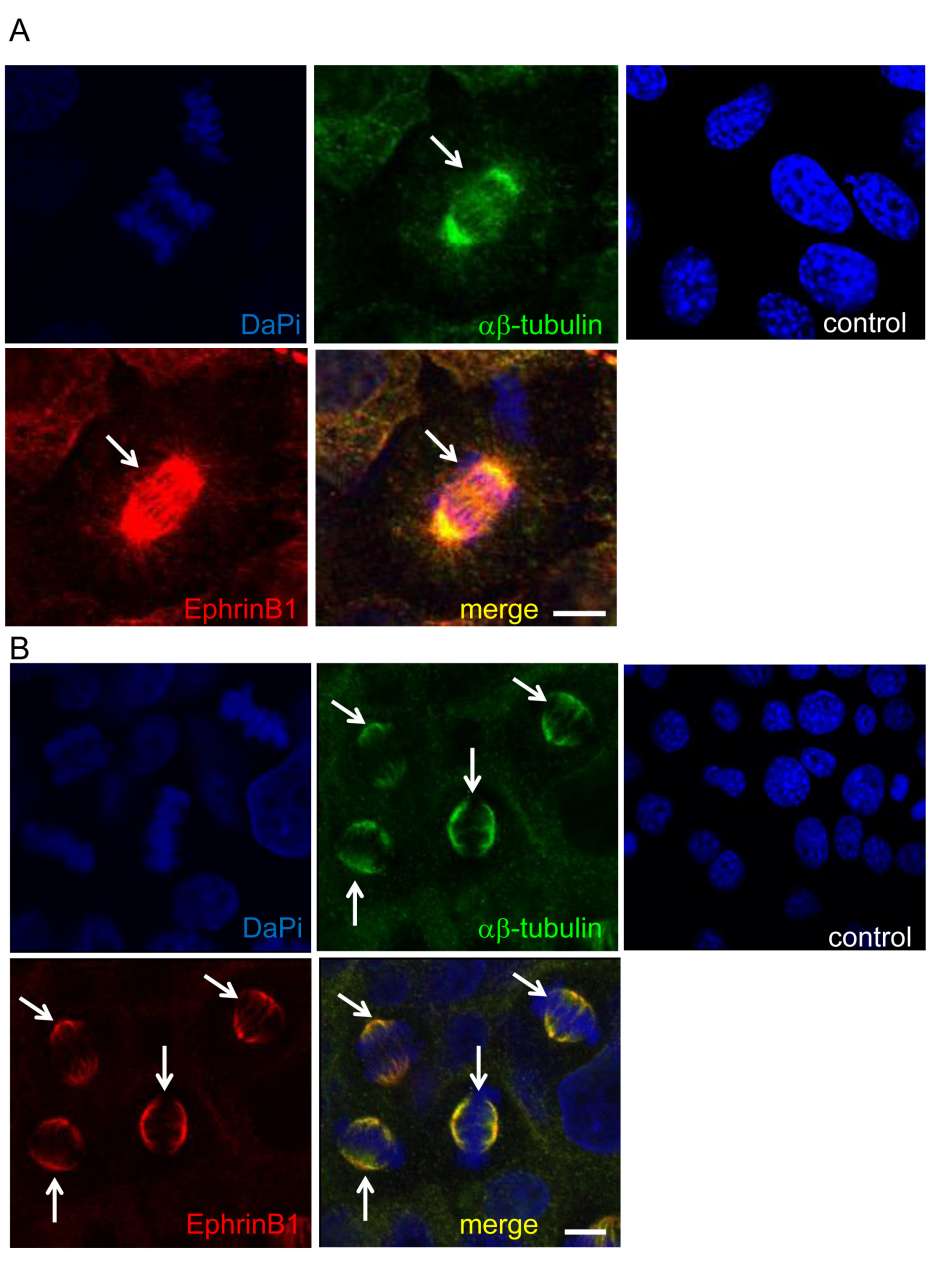
EphrinB1 co-localizes with alpha and beta tubulin Human SCC1 (HPV-) (A) and SCC47 (HPV+) (B) cells were processed for immunofluorescence localizing alpha-beta tubulin (green) and EphrinB1 (red) which co-localize (yellow). Co-localization is particularly robust in mitotic cells, labeling the mitotic spindle (arrows). Scale bar, 10 um. DaPi (blue), nuclear counterstain. Cells in which primary antibody was omitted are labeled “control.”

The focus of the laboratory is head and neck squamous cell carcinoma (HNSCC). Thus, to assess the localization of EphrinB1 during the cell cycle, the human squamous cell carcinoma cell line, SCC1, was processed for co-immunofluorescence (IF) with an antibody recognizing alpha and beta tubulin and one specific for EphrinB1. All cells express EphrinB1 (red) which co-localizes (yellow, merge) with alpha/beta tubulin (green) (Figure 1A). Importantly, EphrinB1's mesh-like localization pattern at interphase is reminiscent of the MT network and co-localizes with alpha/beta tubulin staining (Figure 1A, merge, yellow). However, cells in mitosis show robust EphrinB1 staining of the mitotic spindle (arrows).

**Figure 2:**
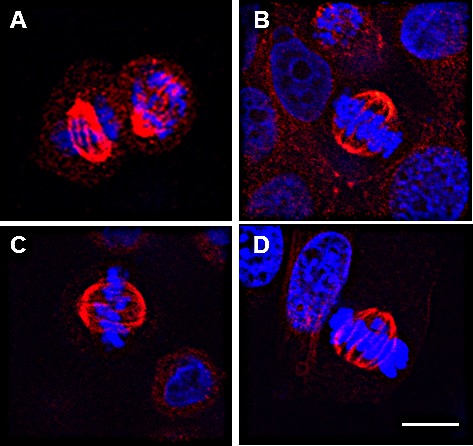
EphrinB1 immunolocalizes to the mitotic spindle in human and mouse cells HeLa cells stained with a rabbit anti-EphrinB1 antibody from AnaSpec demonstrating mitotic spindle staining (red) with a different EphrinB1 antibody than used in Figure 1(A). MCF-7 (B) and MDA-MB468 (C) cell lines were processed for immunolocalization of EphrinB1 (red). EphrinB1 also localizes to mitotic spindles in a mouse model of HPV+ HNSCC, MEERL cells (D). Scale bar, 10 um. DaPi (blue), nuclear counterstain.

Twenty-five per cent of HNSCCs are caused by infection with high risk human papillomavirus (HPV). Thus, to determine whether EphrinB1's pattern of localization changes with HPV infection we next asked whether HPV positive (HPV+) SCC47 cells showed a similar pattern of staining. Like to the HPV negative (HPV-) SCC1 cells, SCC47 cells demonstrate EphrinB1 expression (Figure 1B, red) which also co-localizes with alpha/beta tubulin (Figure 1B, green; merge, yellow) and strongly labels the mitotic spindle (Figure 1B, arrows). The specificity of staining was verified by omission of primary antibodies which resulted in a lack of staining (labeled as control for figures 1A and B). In addition, to further validate that this pattern of EphrinB1 localization was not an artifact, several anti-EphrinB1 antibodies from different commercial sources were used for IF with similar results. An example is given in figure 2A where HeLa cells are stained with a rabbit anti-EphrinB1 antibody from AnaSpec.

To define whether EphrinB1 localizes at the mitotic spindle in other epithelial cells, additional cell lines were similarly analyzed. Robust EphrinB1 immunofluorescence was evident at the mitotic spindle in two breast cell lines, MCF-7 (Figure 2B) and MDA-MB468 (Figure 2C). In addition, we found that this pattern of staining is not limited to human cells as it is also present in a previously characterized mouse model of HPV+ HNSCC called MEERL cells [33]; similar to human cells, EphrinB1 strongly stains the mitotic spindle in these HPV+ cells (Figure 2D). These data suggest that EphrinB1 localization at the mitotic spindle is similar in human and murine cells.

**Figure 3:**
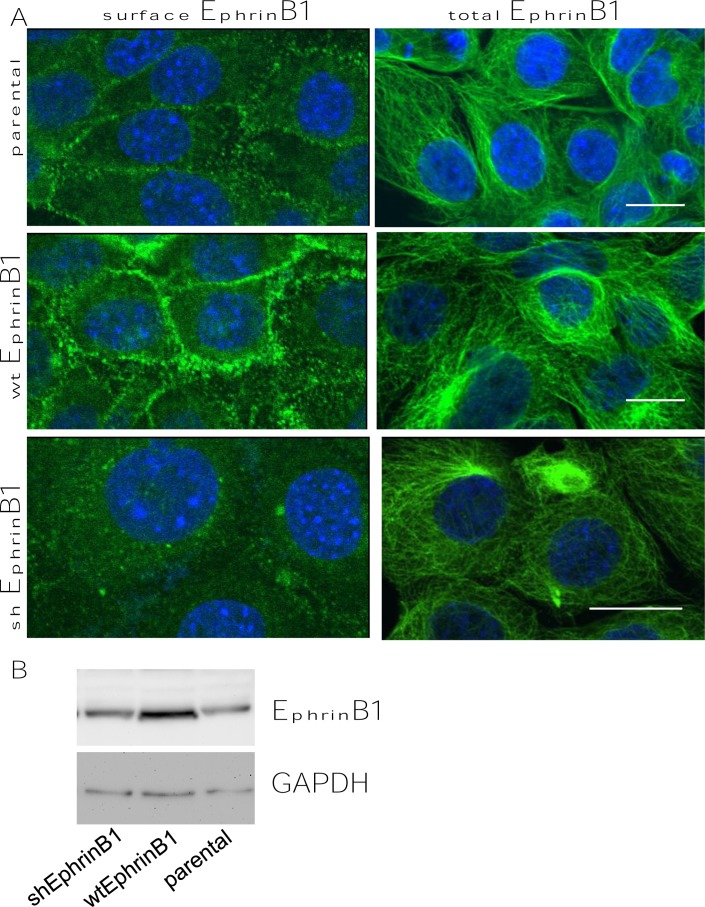
MEERL EphrinB1 stable cell lines Mouse oropharyngeal cells stably expressing HPV16 E6, E7, together with Ras and luciferase (MEERL cells) were used to generate EphrinB1 stable cell lines. Stable MEERL cells expressing wtEphrinB, shEphrinB1 or the parental line were processed for immunofluorescence localizing either surface or total EphrinB1 (seen in green; nuclei counterstained with DaPi, blue) (A). Western blot analysis of these stable cells line demonstrating EphrinB1 expression (EphrinB1) and control for loading (GAPDH) (B).

The most convincing way to demonstrate that EphrinB1 localization at the mitotic spindle is real, would be to generate cells knocked-down for EphrinB1 expression and show loss of spindle localization. Therefore, we generated MEERL cells stably over-expressing (wtEphrinB1); using an EphrinB1 targeting shRNA, we also generated cells stably knocked-down for EphrinB1 expression (shEphrinB1). Several clones were tested with similar results. Surface staining of EphrinB1 demonstrates increased expression in wtEphrinB1 cells relative to the parental cell line (Figure 3A). Interestingly, while surface EphrinB1 protein expression was knocked down in the shEphrinB1 cells relative to the parental cell line (Figure 3A, surface EphrinB1), total EphrinB1(surface and intracellular) was unaffected (Figure 3A, total EphrinB1). Western blot analysis of cell lysates made from these stable cell lines similarly demonstrate that stable over-expression of EphrinB1 (wtEphrinB1) increases total levels of the protein relative to the parental line. However, its stable knock-down (shEphrinB1) fails to show an obvious decrease in total protein expression (Figure 3B). Repeated attempts to generate cells in which both surface and intracellular EphrinB1 were knocked-down were unsuccessful. These data suggest that while cells can tolerate loss of surface EphrinB1 expression, they cannot tolerate its complete loss. This observation is consistent with those of Davy *et al* in their study of the EphrinB1 null mouse [34]. In that study, live EphrinB1 null mice were not recovered in the expected Mendelian ratios demonstrating some level of embryonic lethality with loss of EphrinB1. In addition, despite the fact that EphrinB1 is X-linked, hemizygous females (which retain half a dose of EphrinB1) showed the same perinatal lethality as males (with no EphrinB1). These data emphasize the requirement for EphrinB1 in normal development and are consistent with our studies demonstrating that while some EphrinB1 loss can be tolerated (e.g. loss of surface EphrinB1 in shEphrinB1 cell lines or half dose as in hemizygous female mice), complete loss is much less tolerable.

### EphrinB1 co-localizes with gamma tubulin

**Figure 4:**
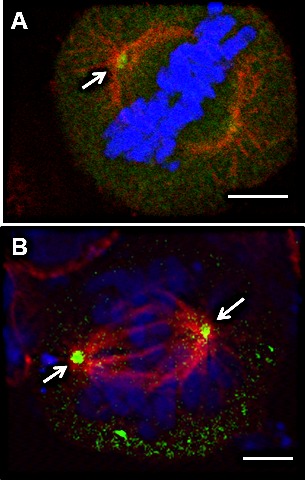
EphrinB1 co-localizes with gamma tubulin at centrosomes MEERL (A) and MDA-MB468 (B) cells were processed for immunolocalization of EphrinB1 (green) and gamma tubulin (red) and found to colocalize at centrosomes (orange, arrows). Scale bar, 2 um. DaPi (blue), nuclear counterstain.

Herrmann *et al* showed that EphrinB1's phosphatase, PTPN13, co-localized with the centrosomal marker, gamma-tubulin [16]. To determine whether EphrinB1 similarly co-localizes with gamma-tubulin, MEERL (Figure 4A) and MDA-MB468 cells (Figure 4B) were processed for IF. Similar to PTPN13, EphrinB1(red) co-localizes with gamma-tubulin (green; merge: yellow, arrows; Figure 4A and B). Taken together, the data suggest that upon entry into mitosis, the majority of EphrinB1 concentrates to the mitotic spindle and also co-localizes with gamma tubulin at centrosomes.

### EphrinB1 staining throughout the cell cycle

**Figure 5:**
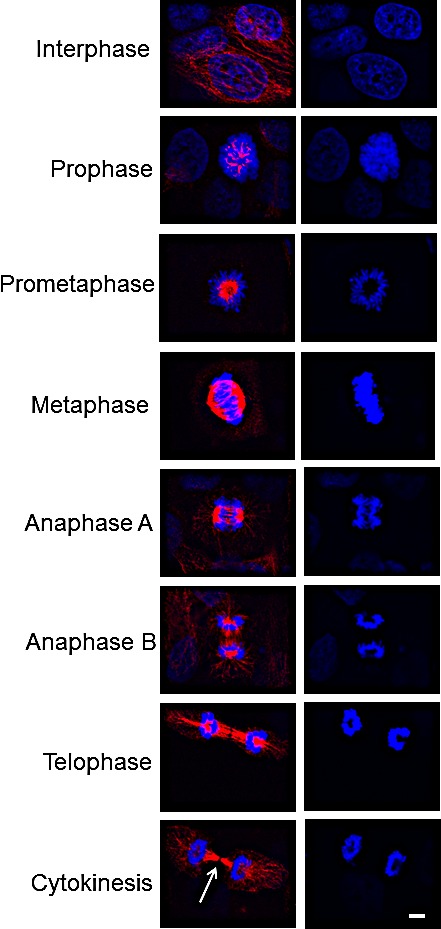
Localization of EphrinB1 throughout the cell cycle SCC1 cells were processed for immunolocalization of EphrinB1 (red) throughout the cell cycle. At cytokinesis, EphrinB1 is missing at the very center of the midbody (arrow). Scale bar, 5 um. DaPi (blue) nuclear counterstain.

EphrinB1's localization at the mitotic spindle prompted us to determine its localization through all phases of the cell cycle. Figure 5 shows that in SCC1 (HPV-) cells, EphrinB1 localizes throughout the cytosol at interphase, concentrating among condensed chromatin in prometaphase, and strongly staining the mitotic spindle from metaphase through anaphase. Towards the end of telophase, EphrinB1 staining again concentrates such that at cytokinesis, robust EphrinB1 staining is found along the midbody except at its very center where EphrinB1 staining is lacking (arrow). Curiously, this point of EphrinB1 absence is exactly the point of localization reported for PTPN13 [16].

### The extracellular domain of EphrinB1 does not localize to the mitotic spindle

**Figure 6:**
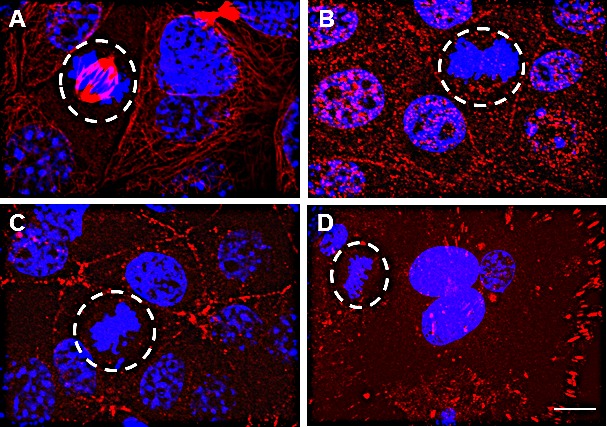
A cleaved, non-phosphorylated fragment of EphrinB1 associates with the mitotic spindle SCC1 cells were processed for immunofluorescence using antibodies for epitope mapping. The mitotic spindle immunostains when using an antibody whose epitope is intracellular (A), however, an antibody whose epitope is extracellular (B) fails to stain the spindle. Antibodies to phospho-EphrinB1 epitopes (Tyr 331, C) (Tyr 317, D) also fail to stain the spindle. Scale bar, 10 um. DaPi (blue), nuclear counterstain.

Full length EphrinB1 (approximately 55kD) is cleaved by matrix metalloproteases in a process known as ecto-domain shedding. This cleavage generates a C-terminal, membrane tethered fragment (CTF, 14-17 kD) which is further processed by gamma-secretase, liberating the intracellular domain (ICD) [35]. Such complex processing suggests that spindle-associated EphrinB1 may not be composed of the full length protein. Therefore, to determine the composition of spindle-associated EphrinB1, SCC1 (HPV-) cells were processed for IF using anti-EphrinB1 antibodies that recognize distinct epitopes. Figure 6A demonstrates staining of EphrinB1 with an antibody recognizing an intracellular epitope which strongly stains the mitotic spindle (red, dotted circle denotes cell in mitosis). In contrast, when cells were stained with an anti-EphrinB1 antibody whose epitope is EphrinB1's extracellular domain, spindle staining was lacking though membrane and cytoplasmic staining were evident as puncta (Figure 6B). These data suggest that the extracellular epitope is not present at the spindle. Figures 6C and D show staining using phospho-specific EphrinB1 antibodies (the antibody used in panel C is specific to phosphorylated tyrosine 331 while the one in panel D is specific to phosphorylated tyrosine 317 of EphrinB1). These antibodies were utilized simply because they were the few commercially available antibodies with phospho-specific EphrinB1 epitopes that worked for IF localization studies. Neither antibody stained the spindle in mitotic cells (dotted circle) though staining at the membrane was evident for each. These data suggest that EphrinB1 phosphorylated on tyrosines 317 or 331 is excluded from the mitotic spindle, consistent with the published literature suggesting that PTPN13 localizes within the spindle and centrosomes [16, 36]. Taken together, these data suggest that full length EphrinB1 is not associated with the mitotic spindle, but that rather a cleaved, unphosphorylated cytoplasmic fragment is the predominant spindle-associated form.

### EphrinB1 staining mitotic figures in human tumor samples

**Figure 7:**
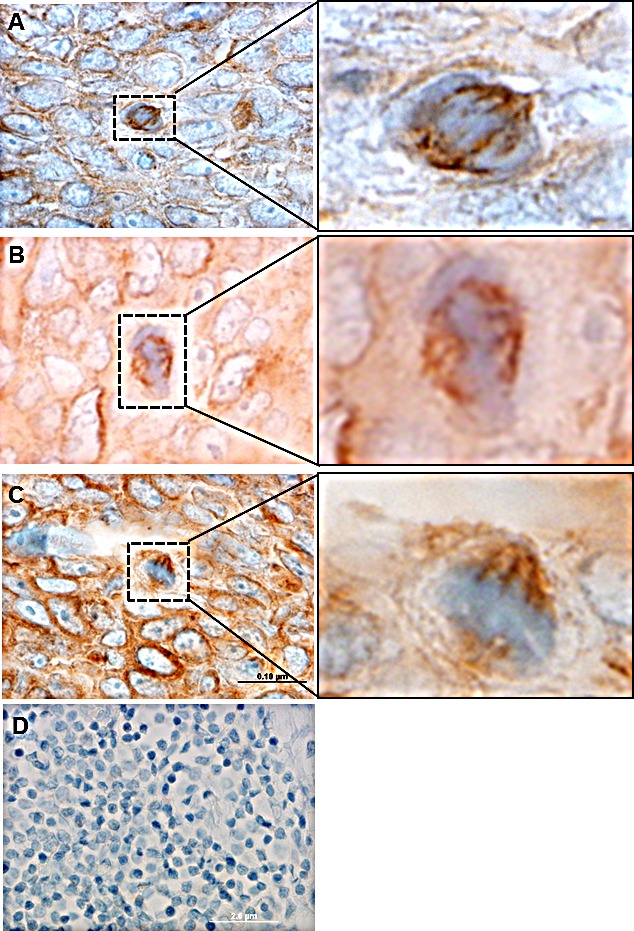
EphrinB1 labels mitotic figures in human tumor Human head and neck tumor sections processed for immunohistochemistry with an EphrinB1 antibody (A-C) show labeling of mitotic spindles (brown). Insets are magnified to show detail. Scale bar, 0.10 um.

While the data presented thus far suggest a potential role of EphrinB1 during mitosis, localization within human tissue would further support a microtubule associated function *in vivo*. To test this, HNSCC tumor sections were processed for immunohistochemistry (IHC) with an EphrinB1 antibody. Figure 7 shows three examples, panels A-C, of mitotic figures (dashed box, and enlarged inset) within tumor that stain the mitotic spindle, similar to that evident *in vitro*. To verify the specificity of antibody staining, tumor sections were pre- incubated with a blocking peptide corresponding to the epitope recognized by the antibody. IHC staining of these sections resulted in a lack of staining (Figure 7D). These data suggest that the mitotic spindle staining evident in cells *in vitro* occurs human tumors *in vivo*.

### Phosphorylated EphrinB1 is excluded from the mitotic spindle

**Figure 8:**
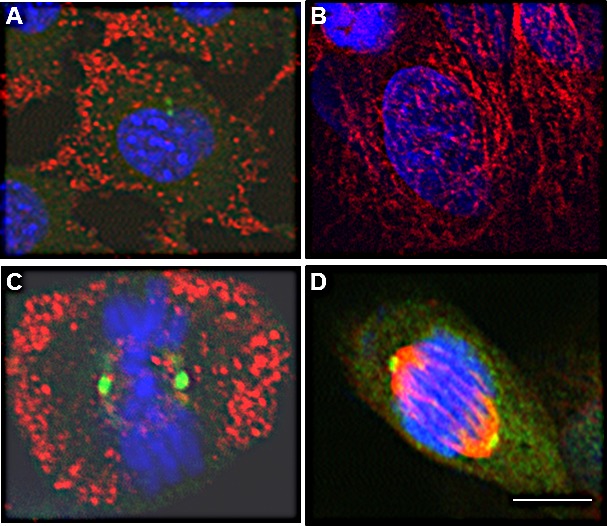
Phosphorylated EphrinB1 is excluded from the mitotic spindle SCC1 cells were processed for immunofluorescence using a phospho-specific EphrinB1 antibody (red) and gamma tubulin (green) at interphase (A and B) and metaphase (C and D). Insets, immunolocalization of EphrinB1 using a non-phospho-specific EphrinB1 antibody (red) and gamma tubulin (green). DaPi (blue, nuclear counterstain.

To further assess the localization of phosphorylated EphrinB1, SCC1 cells were processed for IF with an anti-phosphorylated EphrinB1 antibody that recognizes additional phospho-tyrosines (tyrosine 324 and 329). At interphase, phosphorylated EphrinB1 (red) exists as puncta predominantly at the cell surface but also within the cytoplasm (Figure 8A). This is in stark contrast to the localization of EphrinB1when using an antibody that is not phosphorylation specific (Figure 8B, mesh-like pattern of staining). This antibody's epitope is intracellular. Therefore, it may theoretically recognize cleaved, full length, phosphorylated and non-phosphorylated forms of EphrinB1. Alternatively, the epitope may be best exposed following processing of EphrinB1 and thus it may preferentially bind only cleaved forms of the protein; yet may recognize both phosphorylated and non-phosphorylated forms. Given the significant difference in its staining pattern relative to that of a phospho-specific antibody (Figure 8A), this antibody may recognize predominantly non-phosphorylated forms of EphrinB1 (Figure 8B). During mitosis, phosphorylated EphrinB1(red) becomes largely excluded from the mitotic spindle and does not co-localize with gamma-tubulin (green) or associate with condensed chromatin (blue) (Figure 8C). Again, this localization is striking when compared to EphrinB1's localization when using a non-phospho-specific EphrinB1 antibody (Figure 8D, EphrinB1 red, gamma tubulin green, co-localization, yellow). These data are consistent with PTPN13's co-localization with gamma-tubulin [16].

### A cleaved fragment of EphrinB1 directly binds MTs

The data suggest that a cleaved, intracellular, non-phosphorylated fragment of EphrinB1 associates with MTs. To further validate this finding, the intracellular domain of EphrinB1 (ICD) was cloned and transfected into HEK293 cells. Immunolocalization using EphrinB1 antibodies that recognize intracellular epitopes were used to localize the ICD. Unfortunately, no staining was evident suggesting that these epitopes are conformational and unable to detect the exogenously expressed ICD (data not shown). Thus, an alternative approach was taken to test the binding of EphrinB1's intracellular domain to microtubules. A tubulin spin-down assay was performed. HEK293T cells were transfected with FLAG-tagged-EphrinB1 deleted of its extracellular domain (FLAG-EphrinB1ΔED). Twenty-four hours later cells were harvested, lysed and EphrinB1 was immunoprecipitated (IP) using an anti-FLAG antibody. Bound EphrinB1 protein was recovered via competitive elution with 3X FLAG Peptide and the eluted protein was pre-cleared by centrifugation prior to tubulin binding assays.

**Figure 9:**
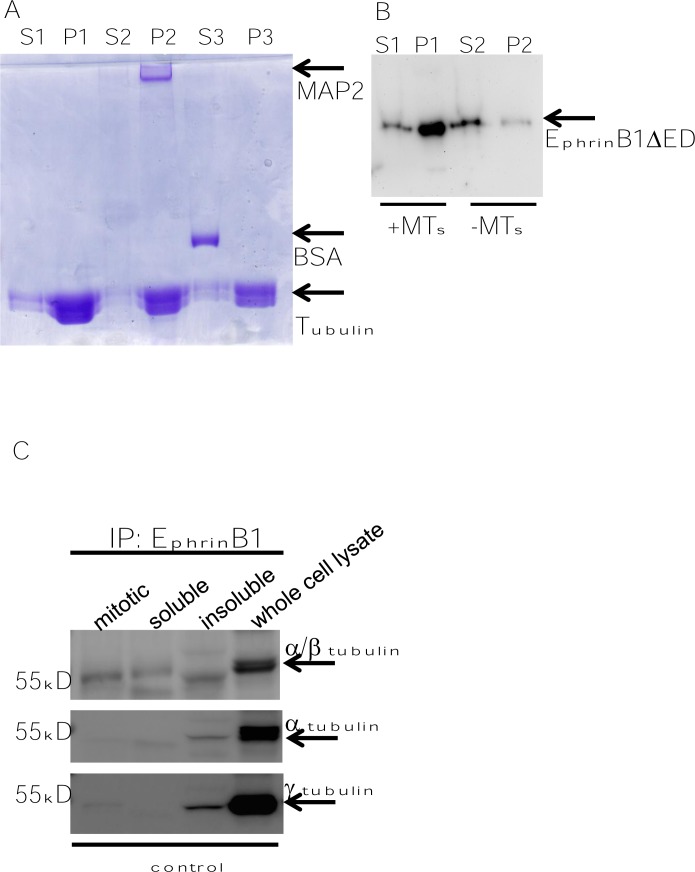
EphrinB1 has novel MAP characteristics and co-immune precipitates with tubulin Coomasie stained SDS-PAGE gel of MAP spin-down assay showing that microtubule associated protein, MAP2, pellets with microtubules but a non-MAP, bovine serum albumin (BSA), does not. S: soluble fraction; P: pellet, 1: microtubules only; 2: MAP2+ microtubules; 3: BSA+ microtubules (A). Western blot analysis of microtubule spin-down of EphrinB1ΔED; S: soluble fraction; P: pellet (B). Western blot analysis of mitotic, soluble and insoluble fractions that have been immunoprecipitated for EphrinB1 (C).

The tubulin spin down assay uses pre-formed microtubules as a substrate to test whether a protein of interest binds to tubulin. Centrifugation separates bound (pellet) and non-bound (supernatant) fractions. As controls, MAP2, a known microtubule associated protein, was added in one condition while bovine serum albumin (BSA), which has no MT binding capacity, was added as a negative control. These samples were centrifuged to separate soluble (S) and pellet (P) fractions which were further subjected to SDS-PAGE and stained with coomasie. Figure 9A demonstrates these control conditions. As expected, tubulin was found predominantly in the pellet fraction (Figure 9A, P1). Similarly, MAP2 was found to pellet with MTs (Figure 9A, P2) while BSA was found exclusively in the soluble fraction (Figure 9A, S3).

To test whether EphrinB1ΔED has MT binding properties, the eluted protein was incubated with or without pre-formed MTs, the samples were centrifuged, separated by SDS-PAGE and analyzed by western blot using an anti-FLAG antibody. Figure 9B demonstrates that in the absence of MTs, the majority of EphrinB1ΔED is found in the soluble fraction (Figure 9B, S2). In the presence of MTs, however, the majority of EphrinB1ΔED is found in the pellet (Figure 9B, P1). These data suggest that EphrinB1 has microtubule binding characteristics.

The interaction between EphrinB1 and tubulin was further assessed biochemically via co-IP of the endogenous protein from SCC1 (HPV-) cells. As fluorescent staining of EphrinB1 exhibited a pattern consistent with mitotic MT's, we included conditions to enrich for dividing cells. SCC1 cells were synchronized to G0 by serum starvation, released from quiescence by serum repletion and incubated further to allow entry into mitosis. At this point, cells were subjected to three rounds of mitotic shake off to harvest mitotic cells. Mitotic cells were collected, lysed and IP'd for EphrinB1. The remaining adhered cells were later lysed and further processed to isolate soluble and insoluble fractions. This additional processing was performed to define where in adhering, non-mitotic cells EphrinB1 resides. Inclusion in the insoluble fraction would suggest that it resides with MTs. In addition, whole cell lysates of SCC1 cells were harvested as an additional control. EphrinB1 was IP'd from each fraction (mitotic, soluble and insoluble) and analyzed by western blot.

EphrinB1 co-IPs with α/β tubulin as well as γ tubulin (Figure 9C). Consistent with the tubulin spin down data, the majority of tubulin (α, β, γ) is present in the insoluble fractions (Figures 1,2,4-8). Interestingly, while α tubulin associates with EphrinB1 in whole cell lysates and insoluble fractions, it is not enriched in mitotic fractions. However, western blot analysis using an antibody that recognizes both alpha and beta tubulin demonstrates co-precipitation with EphrinB1. These data suggest that either EphrinB1 associates with beta tubulin (rather than alpha tubulin) or that it interacts with an epitope made up of alpha and beta tubulin dimers.

### PTPN13 and EphrinB1 expression correlate with sensitivity to taxanes

Chemotherapeutic agents that interfere with mitosis are used clinically to treat many types of cancers; these taxanes either stabilize or destabilize MTs and, in doing so, lead to cell cycle arrest and eventual cell death [37, 38]. One such taxane, paclitaxel, irreversibly binds beta tubulin, promoting MT assembly and interfering with formation and function of the mitotic spindle.

**Figure 10:**
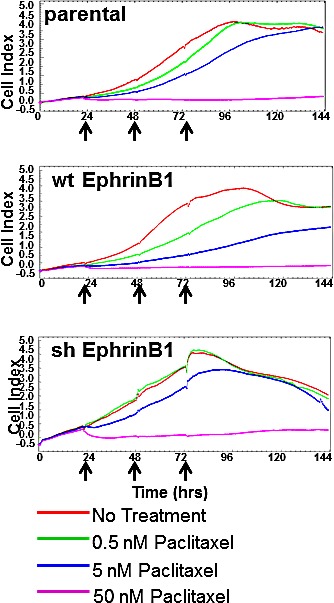
EphrinB1 expression modulates response to paclitaxel Cellular proliferation Xcelligence analysis of MEERL (parental, wtEphrinB1, and shEphrinB1) cells treated with different doses of paclitaxel daily for three days (arrows).

Using different human and mouse cell lines (including MEERL, MDA-MB231, MDA-MB468, HEK293, HaCaT cells), we have previously demonstrated that the absence of PTPN13 expression correlates with increased EphrinB1 phosphorylation [39]. To more directly assess the role of EphrinB1 in mitosis, it was stably over-expressed or knocked-down in MEERL cells (wtEphrinB1 and shEphrinB1 respectively) and tested as follows. Cells were seeded onto microtiter plates and, using the Xcelligence system, their electrical impedance analyzed continuously as an indirect measure of proliferation [40-42]. Different doses of paclitaxel were added every 24 hours (arrows) for 3 days and impedance measured over the course of 6 days. While parental MEERL cells respond in a dose dependent manner to paclitaxel treatment (Figure 10A), the cell index curves shift to the right in wtEphrinB1 cells (Figure 10B) suggesting that over-expression of EphrinB1 renders the cells more sensitive to the drug as compared to the parental line. Conversely, these curves shift to the left in shEphrinB1 cells (Figure 10C) suggesting that knock-down of EphrinB1 correlates with increased resistance to paclitaxel.

Taken together, these data suggest that the ratio of phosphorylated to non-phosphorylated EphrinB1 can alter paclitaxel response such that too little non-phosphorylated EphrinB1 correlates with paclitaxel resistance.

**Figure 11:**
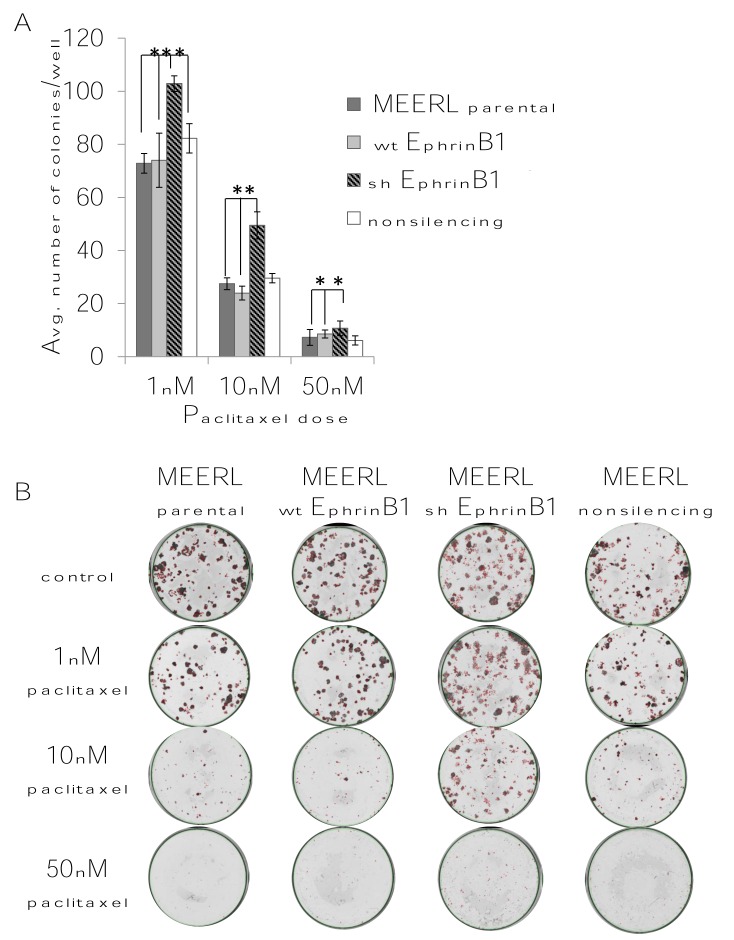
Colony forming assay verification of EphrinB1's modulation of paclitaxel response Colony forming assay quantification (A) and images (B) of MEERL (parental, wtEphrnB1, shEphrinB1) cells treated with different doses of paclitaxel.

As an additional measure of EphrinB1's role in paclitaxel response, a colony forming assay was performed. MEERL parental, wtEphrinB1 and shEphrinB1 cells were again treated with three different doses of paclitaxel for 5 days and the ability of cells to form colonies analyzed (Figure 11). MEERL parental cells demonstrated a dose response to paclitaxel treatment with decreasing colony formation as drug dose increased. A similar trend was evident with MEERL wtEphrinB1 cells. MEERL shEphrinB1 cells were resistant to paclitaxel treatment at all doses tested (p=0.0001 at 1nM, p=0.0008 at 10nM, p=0.002 at 50nM when compared to the parental MEERL cells; p=0.002 at 1nM, p=0.0001 at 10nM and p=0.02 at 50nM when compared to MEERL wtEphrinB1 cells). These data are consistent with those in Figure 10 demonstrating that decreased EphrinB1 expression correlate with increased resistance to paclitaxel treatment.

## DISCUSSION

Our understanding of Ephrin ligand functions continues to increase, demonstrating that these signaling molecules possess a rich variety of functions relevant in health and disease. The present study describes EphrinB1 within the mitotic spindle of both human and mouse epithelial cancer cells. This previously unappreciated localization suggests that EphrinB1 may function as a microtubule-associated protein; alternatively, it may interact with a true MAP indirectly associating with MTs. Consistent with this, we show that EphrinB1 sediments with tubulin and co-localizes with alpha and beta tubulin. In addition, EphrinB1 concentrates at centrosomes co-localizing with gamma-tubulin. Moreover, EphrinB1 localizes at the mitotic spindle upon entry into mitosis where it persists till the end of cytokinesis. Interestingly, we found that during mitosis and cytokinesis, EphrinB1's localization is complementary to that of its phosphatase, PTPN13. Consistent with this, we find that phosphorylated EphrinB1 is predominantly excluded from the spindle suggesting that restriction of EphrinB1-mediated signaling is important, if not required, for proper execution of mitosis and/or cytokinesis. We have previously demonstrated that EphrinB1 signals down the MAP Kinase pathway and that knock-down of EphrinB1 attenuates phosphorylation of Erk1/2 in HEK293 cells [39]. Importantly, strict modulation of Erk1/2 is required for maintaining fidelity of the cell cycle [43, 44]. Thus, our findings that EphrinB1 phosphorylation is regulated throughout the cell cycle are consistent with restriction of Erk1/2 signaling during mitosis. In addition, knock-down of PTPN13 in prostate cancer cell lines increases G0/G1 phase cells and decreases S and G2/M phase cells [19], also suggesting that PTPN13 activity modulates cell cycle progression. With respect to chemotherapeutics, we demonstrate that expression of EphrinB1 in the context of compromised PTPN13 modulates the cellular response to paclitaxel *in vitro*.

Finally, our finding that a cleaved, non-phosphorylated fragment of EphrinB1 directly or indirectly localizes to the spindle while its phosphorylated forms are predominantly excluded from it suggest a mechanism for PTPN13's role in mitosis. For those tumors compromised of PTPN13 expression and/or function, taxane resistance may occur due to lack of EphrinB1 spindle binding. These data suggest that expression of PTPN13 and/or phosphorylated EphrinB1 may function as biomarkers for taxane response. Further studies will define the mechanism of EphrinB1-mediated taxane response and define its function in mitosis. This paper is the first characterization of EphrinB1's role in mitosis and effect on taxane response.

## MATERIALS AND METHODS

### Reagents and antibodies

#### Antibodies used for immunofluorescence

LifeSpan BioSciences rabbit anti-EphrinB1 (LS-C108001, internal epitope), AnaSpec rabbit anti-EphrinB1 (#53460, epitope: residues 136-347), Cell Signaling rabbit anti-alpha/beta tubulin (#2148S), Sigma mouse anti-gamma tubulin (clone GTU-88), R&D Systems goat anti-EphrinB1 (AF473, epitope: K30-S229), Sigma goat anti-EphrinB1 (E5404, extracellular epitope), Santa cruz rabbit anti-phosphotyrosine 317 EphrinB1 (sc-135691), Santa cruz rabbit anti-phosphotyrosine 331 EphrinB1 (sc-153692), LifeSpan BioSciences rabbit anti-phosphotyrosine 329 (human) (LS-C53451).

#### Antibodies used for immunoprecipitation

R&D Systems goat anti-EphrinB1 (AF473, epitope: K30-S229), Sigma mouse-anti-FLAG (#F1804), Anti-FLAG M2 Affinity Gel (Sigma-Aldrich, Cat. # A2220).

#### Antibodies used for western blot

Cell Signaling rabbit anti-alpha/beta tubulin (#2148S), Thermo Scientific mouse anti-alpha tubulin (62204), LifeSpan BioSciences rabbit anti-EphrinB1 (LS-C108001, internal epitope), Sigma mouse-anti-FLAG (#F1804).

Paclitaxel (Sagent) was purchased through Sanford Hospital Pharmacy.

### Cell culture

HEK293, SCC1, 93-VU-147T-UP-C6, MCF7, MDA-MB468 cells were maintained with Dulbecco's modified Eagle medium (DMEM) with 10% fetal calf serum and 1% penicillin/streptomycin. MEERL cells were maintained with E-medium (DMEM/Hams F12, 10% fetal calf serum, 1% penicillin/streptomycin, 0.5 μg/ml hydrocortisone, 8.4 ng/ml cholera toxin, 5 μg/ml transferrin, 5 μg/ml insulin, 1.36 ng/ml tri-iodo-thyonine, and 5 ng/ml EGF).

### Immunofluorescence

Cells were seeded on 8 well chamber slides (Millicell EZ slide, Millipore). At 80% confluence, cells were fixed with 4% paraformaldehyde (EMD Millipore), permeabilized with 0.2% Tx-100 (Pierce), non-specific binding blocked with Superblock blocking buffer (Pierce) and incubated with antibody. Antibody binding was detected with Alexa fluor conjugated secondary antibody (Invitrogen), coverslips mounted with Vectashield plus DaPi mounting medium (Vector Labs) and analyzed with a confocal microscope (Olympus Fluoview 1000).

For surface staining of EphrinB1, cells were again seeded on 8 well chamber slides. When they reached 80% confluence, slides were put on ice for 20 minutes to slow membrane turnover. Cells were incubated with EphB1-Fc (R&D Systems) which consists of the extracellular domain of EphrinB1's cognate receptor (EphB1) fused to human IgG1; this generates a soluble reagent that binds surface expressed EphrinB1. Following washes, cells were then fixed with 4% paraformaldehyde and bound EphB1-Fc detected with anti-human FITC (Sigma, #F9512).

### Generation of EphrinB1 stable cell lines

MEERL cells were previously generated; briefly, oropharyngeal cells from C57Bl/6 mice were isolated and retrovirally transduced to stably express HPV16 E6, E7, Ras and luciferase. MEERL cells were seeded at 40% confluence and mammalian expression plasmid (pcDNA3.1 Zeocin, Addgene) containing either full-length wildtype murine EphrinB1 or an EphrinB1 targeting shRNA were transfected using Lipofectamine 2000 lipid transfection reagent as per manufacturer's instructions (Life Technologies). Following transfection, cells were placed under antibiotic selection with up to 500ug/ml of zeocin (Life Technologies). Untransfected cells died and transfected cells were further ring cloned, expanded and tested for EphrinB1 expression by IF and western blot (examples in Figure 3). At least 40 independent clones for each construct were tested.

### MAP spin-down assay

*In vitro* microtubule binding of FLAG-tagged EphrinB1 was assessed using the Microtubule Binding Protein Spin-down Assay Kit (Cytoskeleton, Inc., Cat. # BK029) according to the manufacturer's recommendations for test protein derived from cell lysates. Briefly, ATCC 293T/17 cells were transfected with p3XFLAG-CMV-7.1-EphrinB1ΔED construct using Lipofectamine 2000 Reagent (Life Technologies, Cat. # 11668). Cells were harvested in 20 mM PIPES (pH 7.0), 2 mM MgCl_2_, 1 mM EGTA, 1 mM GTP, and 0.5 mM PMSF, supplemented with 1X Halt protease inhibitor cocktail. Lysis was achieved by sonication at 4°C using three 15 second pulses at medium power with 1 minute cool down periods between bursts. For each condition, 400 μg of total protein was immunoprecipitated overnight at 4°C using ANTI-FLAG M2 Affinity Gel (Sigma-Aldrich, Cat. # A2220). Bound protein was recovered via competitive elution with a 100 μL volume of 3X FLAG Peptide (Sigma-Aldrich, Cat. # F4799) at a concentration of 100 μg/mL. Eluted protein was pre-cleared by centrifugation at 100,000xG, 4°C, for 20 minutes prior to binding assays. Microtubule assembly and subsequent control and test protein assays were performed according to the standard protocol, in which samples with and without microtubules are floated on top of a cushion buffer (80 mM PIPES pH 7.0, 1 mM MgCl2, 1 mM EGTA, 60% glycerol), centrifuged, and supernatant and pellet fractions isolated and subjected to PAGE. Coomassie blue staining was used for protein detection in control samples. Controls included MAP2, a known microtubule binding protein (positive control) and bovine serum albumin (BSA, negative control) which has no MT binding properties. Test samples (immune-purified FLAG- EphrinB1ΔED, with and without MTs) were transferred to a PVDF membrane (Immobilon-P, Millipore) and probed with an anti-FLAG antibody (Sigma-Aldrich, Cat. # F1804).

### Immunoprecipitation of EphrinB1 and tubulin

Briefly, two 100mm dishes of SCC1 cells were grown to 50% confluence in DMEM supplemented with 10% FBS, at which point cells were washed and incubated in serum-free media to synchronize at G0. After 30h, cells were released from quiescence by repletion of serum and incubated 12h to allow entry into M phase. At this point, one dish was subjected to 3 rounds of mitotic shake-off. Cells removed by shake-off from both treatments were pelleted by centrifugation at 1,000xg, 4°C, for 5m and resuspended in 200μL lysis buffer (50mM Tris HCl pH 7.5, 150mM NaCl, 5mM EDTA, 2mN Na_3_VO_4_, 100mM NaF, 10mM NaPPi, 10% glycerol, 1% Triton X-100) supplemented with 1% Triton X-100 and 1X Halt Protease Inhibitor Cocktail (Thermo 28314 and 78429, respectively). Remaining adherent cells were harvested in lysis buffer, vortexed briefly, and incubated 5m on ice. Insoluble materials were pelleted by centrifugation at 16,000xg, 4°C, for 20m and the soluble (S) fractions removed to pre-chilled tubes. Insoluble pellets were then resuspended in 500μL lysis buffer and both these insoluble (I) fractions, and the mitotic (M) fractions, were sonicated on ice using one 15s burst at medium power. All lysates were immunoprecipitated overnight with 1μg anti-ephrinB1 antibody (R&D Systems, AF473), separated by SDS-PAGE, transferred to PVDF membranes (Immobilon-P, Millipore) and analyzed by western blot using antibodies against α/β tubulin (Cell Signaling 2148S), γ tubulin (Sigma T6557), and α tubulin (Thermo 62204).

### Immunohistochemistry

Head and neck squamous cell carcinoma tumor blocks were sectioned at 5 μm. The BenchMark_®_ XT automated slide staining system (Ventana Medical Systems, Inc.) was used for the optimization and staining. The Ventana iView DAB detection kit was used as the chromogen and the slides were counterstained with hematoxylin. Omission of the primary antibody served as the negative control. In addition, the specificity of antibody staining was verified using an EphrinB1 blocking peptide (Santa Cruz sc-1011P) which eliminated all sample staining.

### Colony forming assay

Cells were seeded on 12-well tissue culture dishes at 200 cells/well. On day 1 (day post-seeding), cells were treated with 1, 10 or 50nM paclitaxel. Untreated cells served as control. All conditions were performed in quadruplicate. Cells were maintained for 5 days at which point they were fixed with 70% ethanol, stained with coomasie blue and colonies counted using the GelCount (Oxford Optronix, United Kingdom).

### Cell Impedence Assay

Five thousand cells/well were seeded on 16 well Xcelligence E plates. Cells received either no treatment (control) or were treated with 0.5nM, 5nM or 50nM paclitaxel every 24 hours for three days. Conditions were performed in quadruplicate and cellular impedance monitored continuously for 6 days. The data are represented as Cell Index (CI), a unitless value derived from the relative change in electrical impedance over time and is used as an indirect measure of cell number. A CI value of zero indicates the absence of cells or their lack of adherence to the electrode.

### Human Samples

All human OSCC patient samples were obtained under written consent and approved by Sanford IRB protocol “Improving the Understanding and Treatment of Head and Neck Cancer.” Parafﬁn-embedded blocks were sectioned and stained using standard immunohistochemical techniques as described above.

### Statistics

Colony counts in each group were obtained from the GelCount (Oxford Optronix, United Kingdom) analyzed using a standard student's T-test.
